# Defective biological networks associated with pseudogene-derived lncRNAs in cancer drug resistance: Promising prospects for their clinical targets in cancer therapy

**DOI:** 10.1016/j.gendis.2025.101728

**Published:** 2025-06-20

**Authors:** Mahsa Aghajani Mir, Abdolreza Daraei

**Affiliations:** a Cancer Research Center, Health Research Institute, Babol University of Medical Sciences, Babol 47176-4774, Iran; b Cellular and Molecular Biology Research Center, Health Research Institute, Babol University of Medical Sciences, Babol 47176-4774, Iran; c Department of Medical Genetics, School of Medicine, Babol University of Medical Sciences, Babol 47176-4774, Iran

**Keywords:** Cancer, Drug resistance, lncRNA, Pseudogene, Pseudogene-derived lncRNA

## Abstract

Cancer is a major cause of mortality globally, characterized by its multifactorial nature and intricate treatment procedures. The main treatment options include targeted drug therapies and chemotherapy. However, overcoming drug resistance remains a significant challenge in curing cancer patients. In recent decades, substantial efforts have been made to explore the resistance of cancer cells to anti-cancer agents and to create methods to counteract this resistance. Cancer cell resistance can be attributed to various factors, including long non-coding RNAs (lncRNAs) involved in cell cycle dysregulation, abnormal DNA repair, cell proliferation, epithelial–mesenchymal transition, metastasis, apoptosis, autophagy, drug efflux transporters, epigenetic modifications, and the formation of cancer stem cells. Pseudogenes are genomic regions that harbor impaired or dysfunctional versions of genes. Although pseudogenes were traditionally considered non-functional, a growing number of them are now being found to serve important biological functions. Recent research has demonstrated that mutations and dysregulation of pseudogene-derived lncRNAs are linked with various human diseases, such as cancer drug resistance. This review concentrates on exploring the latest discoveries that elucidate the diverse molecular functions of regulatory pseudogene-derived lncRNAs implicated in cancer drug resistance and the therapeutic possibilities for overcoming drug resistance.

## Introduction

Cancer represents a major risk to human health and places a considerable strain on society in both wealthy and less affluent nations, remaining the primary cause of new cases and deaths globally.^1^, ^2^, ^3^ In 2022, GLOBOCAN reported that there were around 20 million new cases of cancer diagnosed globally, leading to approximately 9.7 million deaths attributed to the disease.^3^ Moreover, from an impression of this report, which indicates that about 20% of both men and women will be diagnosed with cancer at some point in their lives, while roughly one in nine men and one in twelve women will succumb to the disease.^3^

**Figure 1:**
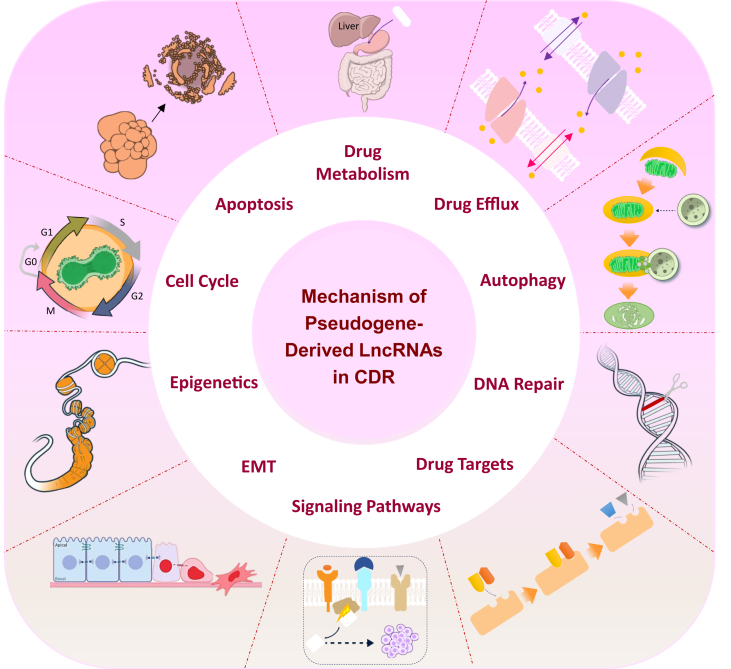
Overview of reported mechanisms of pseudogene-derived lncRNAs in cancer drug resistance. Cancer cell resistance to anti-cancer agents is a multifactorial issue, with genetic differences in individuals, particularly in tumoral somatic cells, and acquired resistance being significant contributing factors. Acquired resistance can arise due to drug efflux and alterations in drug metabolism, while other mechanisms such as alterations in drug targets, DNA damage repair, deregulation of apoptosis and autophagy, resistance-promoting adaptive responses, alterations in the tumor microenvironment, epigenetic changes, epithelial-to-mesenchymal transition and metastasis, cancer stem cells, and inherent cell heterogeneity can also play a role. These mechanisms can occur independently or in combination, making cancer drug resistance a complex and challenging issue (Adapted from NIH BIOART: https://bioart.niaid.nih.gov).

Cancer progression is a complex process characterized by distinct biological features, including aberrant cell growth and differentiation, significant molecular diversity, and epithelial-to-mesenchymal transition (EMT).^4^ Since most cancers are detected in the intermediate or advanced stages, the main treatment strategies typically include molecular-targeted drug therapy and chemotherapy.^5^^,^^6^ Other treatment options encompass radiation therapy, surgery, immunotherapy, and hormonal therapy. Radiation therapy plays a crucial role in cancer care, with around 50% of cancer patients undergoing this treatment at some point during their illness. It accounts for approximately 40% of all curative cancer treatments.^5^^,^^6^ While it effectively targets and eliminates tumor cells, there is also a risk of damaging surrounding healthy tissues. Based on the available evidence, it is reasonable to state that frequently utilized therapeutic agents consist of cisplatin, sorafenib, oxaliplatin, 5-fluorouracil, and epidermal growth factor receptor (EGFR)-tyrosine kinase inhibitors.^7^ Cancer cells can develop resistance to anti-cancer treatments due to several factors, including unique genetic variations, especially within the tumor's somatic cells. Additionally, cancer drug resistance is often acquired and can occur through multiple mechanisms, such as efflux and altered drug metabolism, altered drug targets, DNA damage repair, deregulation of apoptosis and autophagy, adaptive responses promoting resistance, alterations in the tumor microenvironment, epigenetic changes, EMT and metastasis, cancer stem cells, and inherent cell heterogeneity, or a combination of these mechanisms (Fig. 1).^8^, ^9^, ^10^ The prevailing view is that combination therapy should be the optimal treatment approach, as it is expected to prevent the emergence of drug resistance and be more effective than any single drug alone.^9^

In the context of drug resistance, epigenetic modifications represent one mechanism. Epigenetics pertains to an “heritable” phenomenon where phenotypic changes occur independently of the DNA sequence,^11^ suggesting changes in gene expression that are reversible without directly altering the DNA sequence.^12^ Epitranscriptomics, also known as “RNA epigenetics”, is a field of epigenetics that encompasses the modification of RNA and the regulation of ncRNAs.^11^ Long non-coding RNAs (lncRNAs) are the focus of significant research as potential therapeutic targets for overcoming drug resistance in cancer. This interest stems from their role in multiple processes such as cell proliferation, metastasis, EMT, drug efflux transporters, autophagy, cell cycle dysregulation, DNA repair abnormalities, and the formation of cancer stem cells.^10^^,^^13^ lncRNAs engage in various biological processes through their interactions with target sites in either the nucleus or cytoplasm, assuming multiple roles such as chromatin regulators, enhancers, ncRNA sponges (including miRNAs), and molecular scaffolds. lncRNAs play a role in the development of acquired resistance to chemotherapy in cancer. Frequent alterations in lncRNA expression are observed in tumors, leading to diverse patterns of gene expression that contribute to drug resistance.^14^, ^15^, ^16^ As a result, gaining insight into the evolving roles and mechanisms of lncRNAs in drug resistance can create opportunities for developing targeted therapies for cancer patients.

The global burden of cancer remains large, with drug resistance being a major barrier to cancer treatment. The problem of drug resistance in cancer has many resemblances to the field of infectious diseases, as both fields face the challenge of a rapidly increasing number of endogenous and exogenous aggressors.^17^^,^^18^ Tumor resistance can be classified into two categories: intrinsic and acquired. Intrinsic resistance is present before treatment commences, while acquired resistance develops during treatment due to various adaptive responses triggered by the therapy itself.^19^ Nevertheless, prolonged therapies often result in acquired drug resistance and a bleak prognosis.^11^ Acquired drug resistance signifies a newly developed resistance to an initially effective treatment approach.^20^

The growing prevalence of drug-resistant cancers is a major contributing factor to the failure of cancer treatments.^21^ These drug-resistant cancers impose a significant financial burden on both the healthcare system and individual patients. While our current understanding of resistance to targeted cancer therapies is still incomplete, there is a pressing need for innovative and creative approaches to drive further advancements in cancer research and drug development.^22^ This review takes a different approach compared with previous works that have primarily focused on lncRNAs and miRNAs related to cancer drug resistance. Instead, it explores the latest discoveries regarding the various molecular roles of regulatory lncRNAs derived from pseudogenes and the external factors that contribute to drug resistance in cancer. We have outlined the biological significance, regulation, and roles of pseudogene-derived lncRNAs and miRNAs in cancer drug resistance, with a specific emphasis on their interactions and functions. Moreover, we have explored the potential of these pseudogene-derived lncRNAs and miRNAs as targets for cancer therapeutic interventions. Finally, we discuss potential strategies for targeting these pseudogene-derived lncRNAs and miRNAs for cancer treatment and their clinical applications. The emerging perspective is complex, implying that combating clinical cancer drug resistance necessitates extensive research and a more profound comprehension of the underlying mechanisms.

## Multiple drug resistance mechanisms in cancer treatment

Anti-cancer drug resistance is a multifaceted process characterized by diseases developing a tolerance to pharmaceutical treatments.^22^ The concept of drug resistance was initially observed in bacteria, where they developed the ability to withstand the effects of specific antibiotics. Since then, similar mechanisms have been identified in various other diseases, including cancer. Certain types of drug resistance are unique to specific diseases, whereas others, like the active expulsion of drugs, have been conserved through evolutionary processes and are seen in both microbial and human drug-resistant cancers.^9^

Advancements in DNA microarray and proteomics technologies, along with the emergence of targeted cancer treatments, have led to innovative strategies for addressing drug resistance in cancer.^23^^,^^24^ Despite the rapid growth in designing new chemotherapy agents, finding an effective chemotherapy agent against advanced stages of cancer, like invasion and metastasis, continues to be a significant obstacle for researchers and clinicians.^8^^,^^22^ The resistance of cancer cells to anti-cancer agents can be attributed to a range of factors, with individual genetic differences, especially within the tumor's somatic cells, being a significant contributor. Drug resistance in cancer can manifest through various mechanisms, each presenting unique challenges in treatment. In the following sections, we will delve into the most prevalent cases of drug resistance in cancer, shedding light on the complexities involved in overcoming this critical issue.^8^

## Tumor heterogeneity

Tumor heterogeneity refers to differences in cell populations that are not only observed between tumors of the same type from different patients (known as inter-tumor heterogeneity) but also within a single tumor itself (referred to as intra-tumor heterogeneity).^25^^,^^26^ This diversity highlights the complex nature of tumors, where different cells within a tumor or between tumors in different individuals can exhibit distinct genetic, molecular, and phenotypic characteristics.^27^ Tumor heterogeneity is a defining feature of cancer and a significant challenge in the field of cancer research. It is a primary driver of drug resistance, which often leads to the failure of cancer treatments. Mechanistically, tumor heterogeneity can directly impact therapeutic targets or modify the tumor's surrounding environment by shaping the transcriptomic and phenotypic profiles, thereby influencing drug resistance.^26^ Tumor heterogeneity grows both spatially and over time during tumor development, conducting continued reprogramming of the tumor microenvironment. This evolution is also the origin of clones with metastatic potential.^26^ The evolutionary environment of tumors, which is composed of diverse tumor cell populations, presents a significant challenge to effective cancer treatment. All genetically and phenotypically distinct subgroups within a tumor must be effectively eradicated by treatment. If even small surviving subgroups remain, they can lead to repopulation and the development of resistant tumors.^28^ The clinical pattern of acquired resistance in many cases reflects the proliferation of resistant cell clones that may have been present in the original cancer at low levels, but have expanded due to the selective pressure exerted by targeted therapies.^25^ Understanding and addressing this heterogeneity is crucial in developing effective personalized treatment strategies in oncology, as it is essential to target all genetically and phenotypically distinct subpopulations within a tumor to achieve successful treatment outcomes and prevent the development of drug resistance.^25^ This study builds on previous research, suggesting that clonal heterogeneity may contribute to the clinical patterns of acquired resistance to targeted therapies in various cancers, including gastrointestinal stromal tumors, chronic myeloid leukemia, and EGFR-mutant non-small cell lung cancer (NSCLC).^25^

## Stemness

Stemness is an important factor in resistance to therapy, one of the many resistance mechanisms against cancer treatment. Cancer stem cells, also referred to as tumor-initiating cells, are a subset of tumor cells that possess the unique ability to regenerate and differentiate into various cancer cell lineages. These cells are naturally resistant to conventional cancer treatments, which contributes significantly to cancer recurrence.^29^^,^^30^ Within the tumor microenvironment, there are multiple pre-existing clones of cancer stem cells, some of which can adjust and multiply in reaction to alterations in the TME or challenges posed by treatments like radiotherapy and chemotherapy.^31^^,^^32^ The resistance displayed by cancer stem cells is shaped by a blend of intrinsic factors, including quiescence, distinct morphology, DNA repair capabilities, and the overexpression of antiapoptotic proteins, drug efflux transporters, and detoxifying enzymes. Moreover, external factors contribute to the therapy resistance facilitated by cancer stem cells.^29^^,^^30^ The close relationship between tumor drug resistance and the intrinsic or acquired properties of cancer stem cells is significant. These cells can remain dormant in patients who are recovering from cancer treatment, or they can migrate to other organs, ultimately leading to the recurrence of the cancer.^33^ There is an expanding body of literature confirming the presence of cancer stem cells in solid tumors.^31^ Consequently, it is essential to identify and eliminate these small populations of cancer cells to overcome drug resistance and enhance the effectiveness of cancer treatments.

## Drug efflux and alterations in drug metabolism

Anti-cancer drug resistance is a complex phenomenon, with several mechanisms contributing to its development.^34^ The various mechanisms can be generally divided into three main categories: those that depend on the drug, those that depend on the target, and those that are independent of both the drug and the target.^34^^,^^35^ Drug-dependent multidrug resistance is mainly caused by cancer cells producing excessive amounts of efflux pumps and detoxifying enzymes. This reduces the number of drugs that enter the cells and increases the amount that is pumped out.^34^^,^^35^ As a result, the concentration of drugs inside the cells decreases, making anti-cancer treatments less effective. In contrast, target-dependent multidrug resistance is caused by factors that impact the ability of drugs to reach and interact with their intended targets. This includes the target being moved to a different location, deleted entirely, altered through mutation, or produced in excess. Any of these changes can reduce the effectiveness of drugs by preventing proper binding to the target. Finally, drug/target-independent multidrug resistance is caused by changes in the cell's signaling pathways that reduce the impact of drug targeting, regardless of the drug or target involved, which can be genetic or epigenetic.^34^^,^^35^

The protein P-glycoprotein (P-gp) and other drug-pumping transporters are widely acknowledged as key contributors to the development of chemotherapy resistance in cancer cells.^34^^,^^36^ These transporters, which include P-gp/ATP binding cassette subfamily B member 1 (ABCB1), multi-drug resistance protein 2 (MRP2)/ATP-binding cassette subfamily C member 2 (ABCC2), and breast cancer resistance protein (BCRP)/ATP-binding cassette subfamily G member 2 (ABCG2), along with other members of the MRP/ABCC family, play a crucial role in removing anti-cancer drugs from cells. By doing so, they lower the concentration of these drugs inside the cells, reducing the effectiveness of chemotherapy treatments.^34^^,^^37^ Moreover, Gregorcyk et al found that breast cancer patients who tested positive for P-gp had a significantly higher risk of experiencing a relapse.^38^ In hematological cancers, the expression of P-gp mRNA and its protein function increases after chemotherapy. In adult patients with acute myeloid leukemia, the presence of P-gp in myeloid blasts is linked to treatment failure and shorter survival rates.^39^^,^^40^ Clinically, high levels of ABCG2 expression have been observed in various solid tumors, particularly in adenocarcinomas of the digestive tract, endometrium, lung, and breast, and melanoma.^41^, ^42^, ^43^ Furthermore, researchers found^44^ that ABCG2 was overexpressed in 33% of patients with acute myeloid leukemia, and this overexpression was significantly linked to shorter disease-free survival and an increased risk of relapse.^45^, ^46^, ^47^

Anti-tumor drug resistance is driven by several changes, including i) increased activity of drug efflux pumps, including the ABC superfamily, which reduces the intracellular concentration of drugs; ii) decreased drug influx, which limits the amount of drug that can enter the cell; iii) activation of DNA repair mechanisms, which allows cancer cells to improve the chemotherapy impairment; iv) metabolic modification or detoxification, which allows cancer cells to neutralize the impacts of anti-cancer drugs; v) altered expression of apoptosis-associated protein B-cell leukemia/lymphoma 2 protein (Bcl-2)^48^ and tumor suppressor protein p53,^49^ which can inhibit programmed cell death and promote cancer cell survival.^50^ A key factor leading to multidrug resistance in tumor cells is the excessive production of ABC transporters. These transporters use ATP-driven energy to pump both toxic substances and cancer-targeting medications out of the cells, thereby reducing their intracellular concentration and effectiveness.^34^^,^^51^^,^^52^

Initially identified in cancer cells that had developed resistance to chemotherapy, membrane drug transporters were found to actively transport drugs across cell membranes, ejecting them from the cell. Subsequently, these transporters have been discovered in different healthy tissues, where they serve a protective function by preventing the accumulation of lipophilic xenobiotics that could otherwise penetrate cell membranes unchecked.^53^ Researchers have explored the development of inhibitors that target ABC efflux transporters, with the goal of using them as chemosensitizers to counteract resistance.^34^ However, clinical trials have shown that numerous chemosensitizer compounds are toxic and offer little to no advantage for cancer patients. Consequently, novel inhibitor candidates are currently undergoing further research and investigation. Recent studies indicate that the efflux pumps belonging to the ABC transporter family are influenced by epigenetic regulation mechanisms.^34^

## Apoptosis

Cell death is a critical component of normal development and maturation. Maintaining a healthy balance between cell proliferation and cell death is necessary for preserving regular physiological functions. However, defective or inadequate apoptosis is a characteristic acquired by cancer cells.^54^^,^^55^ Apoptosis is a highly organized and pre-programmed process of cell death. It can be initiated via two primary pathways: the extrinsic pathway, which is activated by the stimulation of death receptors on the cell membrane, and the intrinsic pathway, which begins with a sequence of cellular events primarily taking place in the mitochondria.^56^^,^^57^ Both the extrinsic and intrinsic pathways lead to the coordinated and controlled process of apoptosis within the cell. Apoptosis has been shown to be crucial in tumor development and cancer therapy. Defects in the process of apoptosis can result in the proliferation of a population of neoplastic cells.^56^ Proteins that participate in the intrinsic pathway of apoptosis, such as those from the Bcl-2 superfamily, inhibitors of apoptosis (IAP), and the tumor suppressor p53, have been linked to the emergence of multidrug resistance in different types of cancer.^58^

The external pathway of apoptosis involves several ligands and cell death receptors, including Fas receptor (FAS) and tumor necrosis factor receptor (TNF-R), as well as linker proteins and caspase-3/6/7/8. This pathway leads to the degradation of actin and nuclear lamin proteins, ultimately causing cell apoptosis.^59^ In the intrinsic pathway that takes place in the mitochondria, specific proteins have distinct functions. Anti-apoptotic proteins, such as Bcl-2 and protein kinase B (PKB or AKT), counteract cell death, while pro-apoptotic proteins like Bcl-2-associated protein x (Bax), Bcl-2 antagonist killer 1 (Bak), and caspase-9 promote it. In tumor cells, an increase in anti-apoptotic genes like Bcl-2 and AKT, along with a decrease in pro-apoptotic genes such as Bax and B-cell lymphoma-extra-large (Bcl-xL), is associated with increased resistance to chemotherapy.^60^ Mutations in the pro-apoptotic p53 gene can contribute to drug resistance. Normally, p53 is essential for triggering apoptosis in response to cellular stress and DNA damage from chemotherapy. However, inactivating mutations in p53 can sever the connection between DNA damage and the activation of apoptosis, resulting in heightened resistance to chemotherapy.^61^ The phosphatidylinositol 3-kinase (PI3K)/AKT signaling pathway plays a crucial role in promoting the survival and growth of cancer cells, and it is frequently overactive in tumors that demonstrate multidrug resistance. Additionally, the tumor microenvironment, especially factors released by cancer-associated fibroblasts, can suppress apoptosis in cancer cells and reduce the effectiveness of different anti-cancer treatments.^58^ Some research indicates that the progression of various cancers, including B-cell lymphoma,^62^ breast cancer,^63^ and pancreatic cancer,^64^ is linked to the dysregulation of apoptosis inhibitors.

## Autophagy

Autophagy, also known as macroautophagy, is a cellular process that occurs in response to nutrient deprivation or metabolic stress. It is a fundamental mechanism in eukaryotic cells for the degradation of large molecules and organelles.^65^ This process is crucial for conserving cell survival and genetic stability, but it can also play a role in developing resistance to cancer treatments. Comprehending the complex role of autophagy in cancer may enable scientists to develop new strategies to control drug resistance and improve cancer treatment.^66^ In some cases, autophagy can promote chemoresistance and support the survival of cancer cells, making the disease more difficult to treat. On the other hand, autophagy can increase chemosensitivity and facilitate cell death, which can be beneficial in cancer treatment.^67^ Autophagy is involved in controlling the growth and spread of tumor cells. This process is affected by various factors, such as AMP-activated protein kinase (AMPK), mitogen-activated protein kinase (MAPK), PI3K/AKT, beclin 1 (BECN1), and autophagy-related (ATG) proteins. ncRNAs are also important regulators of autophagy, as they can control how cancer cells respond to certain drugs.^67^ Research has shown that inhibiting the autophagy signaling pathway significantly helps overcome chemotherapy resistance and resensitizes tumor cells to anti-cancer treatments. This effect has been observed in various cancer types, including colorectal cancer,^68^ brain cancer,^69^ breast cancer,^70^ and ovarian cancer.^71^ By elucidating the role of autophagy in cancer, scientists may be able to develop new treatments that can help overcome cancer drug resistance.

## Epigenetics

Many research studies have emphasized the significance of epigenetic modifications in cancer drug-tolerant persister cells, which can survive within the diverse tumor population and adapt to become more tolerant to higher levels of drug exposure.^72^ Furthermore, cancer cells can restructure their epigenomic landscape through two primary types of epigenetic alterations: DNA methylation and modifications to histone and non-histone proteins. These changes enable cancer cells to generate various drug resistance mechanisms to circumvent anti-cancer therapies.^73^ Furthermore, other epigenetic regulators, such as DNA methyltransferases, chromatin readers, writers, and erasers, various histone modifiers, and ncRNAs, play a crucial role in precisely regulating gene expression involved in numerous therapy resistance modes.^72^^,^^73^

Recent research has revealed that epigenetic mechanisms, encompassing DNA methylation and histone modification, play a pivotal role not only in regulating the expression of protein-coding genes but also in modulating the activity of miRNAs such as let7a, miR-203, miR-137, miR-148, miR-34a, miR-124, and miR-9. On the other hand, a specific group of microRNAs, including those that target DNA methyltransferases, histone deacetylases, and polycomb group genes, control the expression of crucial epigenetic regulators.^74^, ^75^, ^76^ This intricate interplay between epigenetic pathways and miRNAs forms a complex regulatory circuit, shaping the overall gene expression profile. Disturbance of this regulatory system involving epigenetics and miRNAs can disrupt normal physiological functions and contribute to the development and progression of several diseases.^75^^,^^76^ Comprehending the crosstalk between epigenetic mechanisms and miRNAs is crucial for unraveling the molecular underpinnings of diseases and may offer valuable insights for developing potential treatments focused on restoring proper regulatory control and mitigating disease-associated dysregulation.^74^^,^^75^ By modulating these epigenetic factors, cancer cells can adapt to shifting environmental conditions and acquire resistance to a wide range of anti-cancer treatments, such as ovarian cancer,^77^ colorectal cancer,^78^ and breast cancer.^79^ A comprehensive understanding of these epigenetic mechanisms is critical for creating effective new treatments that can counteract drug resistance and enhance patient outcomes in cancer care.

According to the current paradigm, combination therapy is considered the optimal treatment approach. This strategy is believed to inhibit the outreach of drug resistance and demonstrate greater efficacy compared with individual drugs used in isolation.^9^ The rationale behind this approach lies in the synergistic effects of combining multiple drugs, targeting different pathways or mechanisms, to enhance treatment outcomes and overcome the challenges posed by drug resistance. By utilizing a combination of therapies, clinicians aim to maximize treatment effectiveness while minimizing the likelihood of resistance development, ultimately improving patient outcomes in the management of different illnesses, including cancer.

## Pseudogenes: identification and structure

The rapid advancements in bioinformatics analysis and the next-generation sequencing technology for whole genomes and transcriptomes have revealed that only 2% of the human genome is responsible for encoding proteins, while the remaining 98% of transcriptional products are ncRNAs.^80^ Over the last ten years, the significance of nonprotein-coding functional elements in the human genome has surfaced and been recognized as a pivotal discovery in post-genomic biology. Following the completion of large-scale projects like the Encyclopedia of DNA Elements (ENCODE) and Functional Annotation of Mammals (FANTOM), 16,000 pseudogenes, along with numerous lncRNA genes, have been identified.^81^ Recent experimental data suggest that while 15,000 pseudogenes have been identified, only around 10% of these are actually transcribed. Moreover, approximately 19% of identified human lncRNAs are derived from the transcription of pseudogenes.^82^^,^^83^ Molecular functions for only a handful of these pseudogenes have been characterized.^84^

Pseudogenes are copies of genes that have accumulated various mutations over time, causing them to lose their original function. These mutations may involve frameshift changes, the occurrence of premature stop codons, and the repositioning of genes to inactive heterochromatin areas within the genome.^85^ Initially, pseudogenes were considered to be non-functional genetic material that resulted from gene mutations that inactivated them throughout evolution. However, some recent reports have indicated that the dysregulation of pseudogenes is linked to a variety of physiological and disease processes, including cancer.^86^^,^^87^ The term “pseudogene” was first coined by Jacq et al in 1977 when they identified a copy of the 5S rRNA gene in *Xenopus laevis*. This copy had a truncated 5′ end and 14-base pair mismatches, rendering it non-functional.^88^ Subsequently, numerous pseudogenes have been progressively identified in various prokaryotes and eukaryotes, including humans. Historically, pseudogenes were considered as non-functional “junk DNA”, “relics of evolution”, or “genomic fossils”.^88^

Pseudogenes can generally be divided into two primary categories based on their structural features and origin: processed and unprocessed. Processed pseudogenes are created when cDNAs, which are produced by reverse transcribing parental genes, get integrated into new locations in the genome. Consequently, processed pseudogenes lack introns.^89^^,^^90^ Most of these molecules exhibit a poly(A) sequence at their 3′ end due to mRNA 3′ end polyadenylation. Additionally, these pseudogenes are bordered by duplicated integration sites that vary in length from 5 to 20 base pairs.^89^ Interestingly, a subset of processed pseudogenes has been found to originate from circular RNA (circRNA) transcription. These pseudogenes derived from circRNAs usually lack a poly(A) sequence at their 3′ end and display an inverted arrangement of introns in relation to the original mRNA sequences.^89^

**Figure 2:**
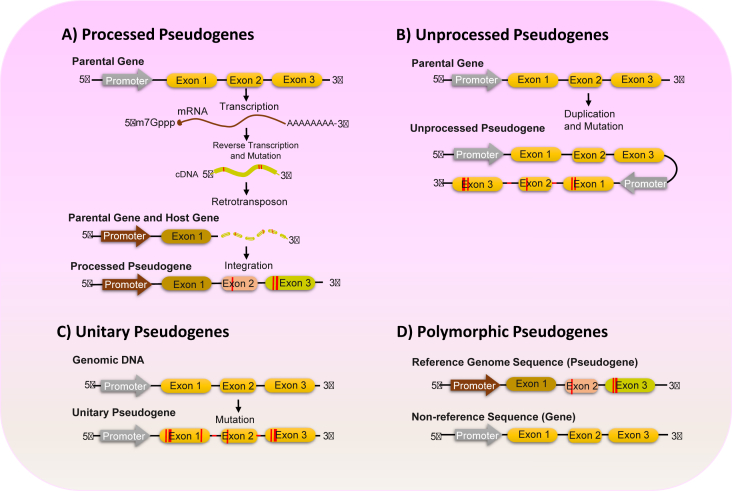
Illustration of various types of eukaryotic pseudogenes. **(A)** Processed pseudogenes originate from the reverse transcription and integration of a processed mRNA by a retrotransposon located on a different chromosome than the original gene. **(B)** Unprocessed pseudogenes arise from gene duplication events that accumulate mutations, hindering their translation, and are situated on the same chromosome as the parental gene. The original gene copy remains fully functional. **(C)** Unitary pseudogenes result from multiple mutations in an ancestral protein-coding gene, leading to the loss of its transcription and translation capabilities. **(D)** Polymorphic pseudogenes are sequences with inactivating mutations in the reference genome but remain intact in other non-reference genomes.

In contrast to processed pseudogenes, the second category includes unprocessed pseudogenes that contain introns in their sequences. These unprocessed pseudogenes can exist in two forms: unitary (orphan) or duplicated. Unitary pseudogenes arise from single-copy functional genes that have accumulated random mutations over time, resulting in the loss of their original functions. Consequently, unitary pseudogenes lack paralogs within the same genome but may possess orthologs in related species.^91^ Moreover, through the process of unequal crossing-over, some genes that undergo tandem duplications can give rise to duplicated pseudogenes. These duplicated genes can then accumulate additional mutations, leading to their transformation into distinct new pseudogenes. Due to their origin mechanism, duplicated pseudogenes are located on the same chromosomes as their parent genes.^92^ A last class of rare pseudogenes includes polymorphic pseudogenes, which contain disabling mutations in the reference genome but remain intact in other non-reference genomes (Fig. 2).^83^

## Pseudogenes: functions

Based on our current knowledge, pseudogenes can influence gene expression through both transcriptional and post-transcriptional mechanisms.^93^ Pseudogenes serve various functions beyond being considered as nonfunctional gene “trash” or “relic”. For instance, at the DNA level, pseudogenes can influence the sequences of their parent or host genes through random insertion or exchange of DNA sequences, which can further impact their structures and functions.^90^^,^^93^^,^^94^ At the RNA level, pseudogenes can produce transcripts that serve important regulatory functions. These pseudogene-derived RNA molecules can act as antisense RNAs, small interfering RNAs (siRNAs), and miRNA decoys, also called competing endogenous RNAs (ceRNAs). In these roles, they can modulate the expression of target genes at the post-transcriptional stage. Finally, at the protein level, pseudogenes may be capable of encoding proteins or peptides that function as “functional” genes involved in gene regulation networks.^90^^,^^93^^,^^94^ Additionally, RNA transcripts from parent genes and their homologous pseudogenes can compete for binding to RNA-binding proteins. This competition can have either a positive or negative effect on the parent gene mRNAs, depending on the specific functions of the RNA-binding proteins involved.^93^^,^^94^ Changes in pseudogene transcript levels can result in alterations to the mRNA levels of the parent gene. This ability allows pseudogenes to function as both positive and negative regulators of gene expression. Consequently, pseudogenes play a crucial role in influencing the human genome under different situations, particularly in disease states.^93^^,^^94^ As evidenced by data from next-generation sequencing analysis, pseudogenes serve another purpose in producing lncRNAs. These transcripts consist of lncRNA molecules that lack protein products, although in some instances, they may generate short peptides. lncRNAs represent a category of ncRNAs with sequence lengths exceeding 200 nucleotides, playing diverse roles in biological processes. These lncRNAs serve various functions, such as regulating transcription, translation, and epigenetic modifications, modulating protein factors and chromatin, guiding specific ribonucleoprotein complexes, and acting as scaffolds for designated ribonucleoprotein assemblies.^95^, ^96^, ^97^

Studies have shown that lncRNAs can serve as both sources and inhibitory regulators of miRNAs. A significant feature of lncRNA biology is the existence of a network of interactions with miRNA pathways that regulate gene expression at the post-transcriptional level.^98^, ^99^, ^100^ It is suggested that lncRNAs act as molecular sponges for miRNAs; for example, lncRNA zinc finger antisense1 (ZFAS1) modulates miR-150-5p in head and neck squamous cell carcinoma.^98^, ^99^, ^100^

Recent research appears to support the idea that, at the transcriptional level, a new model is emerging in which lncRNAs link DNA and proteins by binding to chromatin and serving as scaffolds for modifying protein complexes. Their ability to adopt various secondary structures enables RNA to interact with a diverse array of substrates in a highly specific manner.^98^^,^^99^ This mechanism facilitates the connection between promoters and enhancers or enhancer-like non-coding genes through the regulation of chromatin looping. Additionally, it imparts specificity to histone-modifying complexes by directing them to specific genomic loci to modulate gene expression.^98^

**Figure 3:**
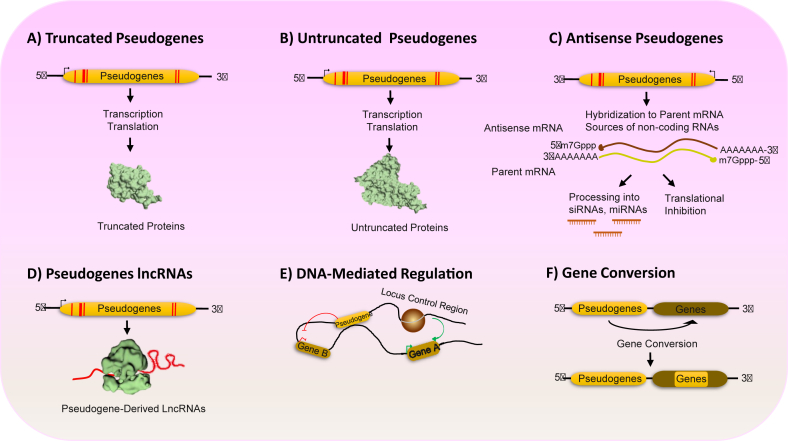
Overview of pseudogene functional mechanisms. **(A)** Some pseudogenes retain or acquire the capacity to encode short peptides or truncated proteins, functioning through intact domains. **(B)** Untruncated pseudogenes may exhibit high homology to parental proteins, encoding full-length proteins closely resembling their parent genes but expressed in a different context. **(C)** Pseudogenes transcribed in antisense produce transcripts complementary to their parental gene mRNAs, potentially regulating the parental mRNA through an antisense mechanism (miRNA) or inhibiting translation (siRNA). **(D)** Pseudogenes can encode long non-coding RNAs (lncRNAs) that operate via RNA-protein interactions or act as competing endogenous RNAs (ceRNAs) for miRNA sponges. **(E)** Pseudogenes can function independently of RNA by facilitating 3D chromatin interactions and influencing host gene transcription through epigenetic silencing. **(F)** Pseudogenes and parental genes can transfer harmful alleles to their parent genes through non-allelic recombination (gene conversion) or homologous recombination.

Further investigation in this field may reveal that lncRNAs have the potential to function as tumor biomarkers and exhibit both tumor-suppressive and oncogenic roles, influencing various aspects of cellular homeostasis such as proliferation, survival, migration, and genomic stability. However, the specific functions of some of these transcripts are still not fully understood.^101^ Recent studies indicate that only a small subset of pseudogene-derived lncRNAs have been identified so far, with limited understanding of their functions. Nevertheless, research in this area is still in its early stages, emphasizing the significance of pseudogene-derived lncRNAs as crucial components of the genome regulatory network (Fig. 3).

## Anti-cancer drug resistance mechanism and involvement of pseudogene-derived lncRNAs

There is overwhelming evidence corroborating the notion that multilayered functions of pseudogene DNAs, RNAs, or proteins have been reported in various types of human cancer,^102^ among which pseudogene-derived RNA transcripts are the most investigated. In the last decade, the category known as pseudogene-derived RNA transcripts has become increasingly prominent in studies related to tumorigenesis.^88^ The improper regulation of pseudogenes and their transcripts has been linked to the onset and progression of multiple human diseases, including cancer. Within pseudogene-derived lncRNAs, some promote tumors, aiding in cancer growth, while others act as tumor suppressors, impeding cancer advancement.^88^ Additional evidence indicates that, much like conventional lncRNAs, pseudogene-derived lncRNAs serve as significant regulators in the initiation and progression of human cancer through the ceRNA mechanism by sponging miRNAs. This assertion is supported by a growing body of research.^88^

The primary theoretical basis for the function of pseudogene-derived lncRNAs is that they may act as antisense RNAs or endogenous small interfering RNAs (endo-siRNAs).^103^ Additionally, recent research has shown that RNAs expressed from pseudogenes can function as miRNA sponges, thereby playing important biological roles. To gain a deeper understanding of the miRNA sponging mechanism associated with pseudogene-derived lncRNAs in cancer, it is essential to introduce the ceRNA mechanism proposed by Salmena et al (2011).^103^

**Figure 4:**
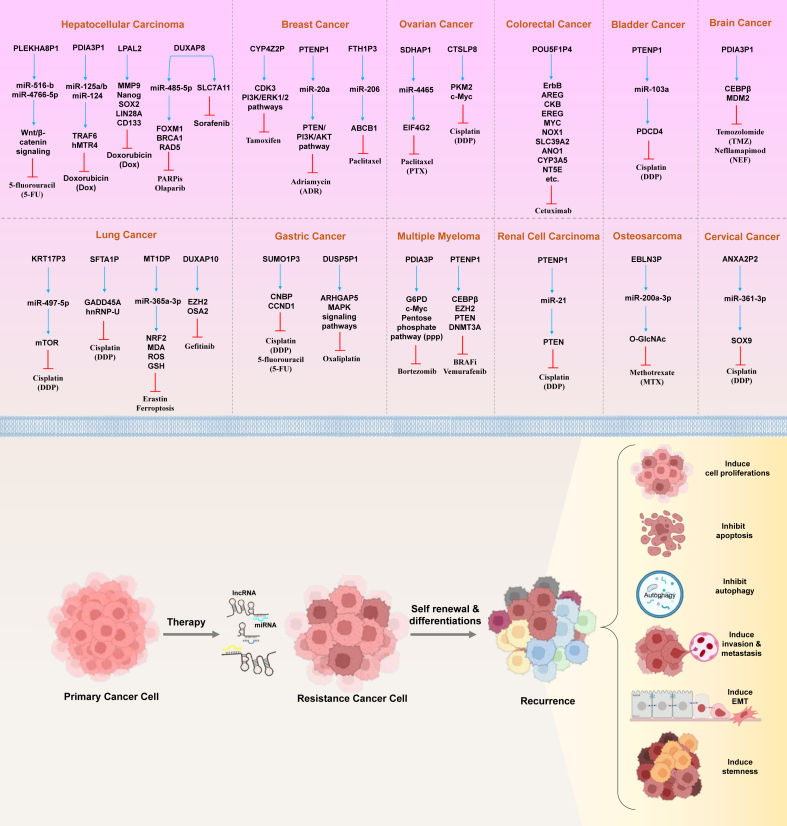
Pseudogene-derived lncRNAs and their miRNA sponging mechanism in cancer drug resistance. According to the studies discussed, pseudogene-derived long non-coding RNAs (lncRNAs) have a significant impact on gene expression and signaling pathways related to drug resistance in cancer. These lncRNAs participate in various biological processes through their interactions with target sites in either the nucleus or cytoplasm, acting as non-coding RNA sponges (including miRNAs). These functions can contribute to the development of acquired resistance to chemotherapeutic drugs in cancer. Most research on the molecular mechanisms of action of pseudogene-derived lncRNAs has focused on the competing endogenous RNA (ceRNA) hypothesis, where they compete for shared miRNAs with either cognate or non-cognate genes. This competition can lead to changes in gene expression and signaling pathways, which can contribute to the development of drug resistance in cancer. Understanding the molecular mechanisms of action of pseudogene-derived lncRNAs is crucial for developing new strategies to overcome drug resistance in cancer treatment (Adapted from BioRender: https://www.biorender.com).

According to this hypothesis, messenger RNA, lncRNA, and circRNA can interact with one another by binding to common miRNAs through miRNA response elements. When lncRNAs, pseudogenes, and circRNAs are dysregulated, it results in changes in miRNA abundance, which in turn affects their ability to inhibit the expression of downstream targets. This mechanism is also relevant for transcripts derived from pseudogenes. Thus far, the ceRNA mechanism has been shown to play a role in the initiation and progression of human cancer when it is dysregulated.^104^^,^^105^ Utilizing the ceRNA mechanism, researchers have identified numerous potential cancer-associated pseudogenes through *in silico* analysis. By examining existing literature and public databases, certain pseudogene-derived RNAs can be classified as predictive, inheritable, or prognostic biomarkers.^106^ Recent investigations have uncovered mutations and dysregulation of pseudogene-derived lncRNAs linked to various forms of cancer drug resistance (Table 1). This section aims to explore recent findings that clarify their role in the molecular mechanisms underlying anti-cancer drug resistance (Fig. 4).

**Table 1:** Summary of dysregulated pseudogene-derived lncRNAs and their molecular mechanism in diverse types of cancer drug resistance.

Cancer	Pseudogene-derived lncRNA	Patients and cells tested	Drug	Affected target	Description	Reference
Hepatocellular carcinoma	PLEKHA8P1	FT3-7, mouse models	5-Fluorouracil	miR-516-b, miR-4766-5p, Wnt/β-catenin signaling	The PLEKHA8P1-PLEKHA8 axis could exert a cytoprotective effect against 5-fluorouracil and promote tumor progression through Wnt/β-catenin signaling.	108
	PDIA3P1	Hepatocellular carcinoma tissue, SK-Hep-1, QGY-7703, mouse models	Doxorubicin	miR-125a/b, miR-124/TRAF6, hMTR4	The regulatory axis of hMTR4-PDIA3P1-miR-125/124-TRAF6, which controls chemoresistance through the NF-κB and hMTR4 signaling pathways, operates via RNA degradation and DNA damage mechanisms.	112
	LPAL2	Hepatocellular carcinoma tissue, HepG2, Hep3B, Huh7, HA22T, Mahlavu, SK-Hep1, mouse models	Doxorubicin	MMP9, Nanog, SOX2, LIN28A, CD133	LPAL2 alleviates doxorubicin resistance and tumor growth, invasion, migration, and stemness phenotypes of hepatocellular carcinoma cell lines. Additionally, down-regulation of LPAL2 in hepatoma cells impairs doxorubicin-induced cell death by modulating the expression of apoptosis-related markers.	113
	DUXAP8	THLE-3, HCCC9810, BEL-7402, HUH-7, SMMC-7721, HepG2; HepG2/ADR, mouse models	PARP inhibitors, olaparib	miR-485-5p, FOXM1, BRCA1, RAD5	The signaling cascade of DUXP8/miR-485-5p/FOXM1 axis regulates proliferation, migration, epithelial-to-mesenchymal transition, and chemoresistance.	117
		Hepatocellular carcinoma tissue, mouse models	Sorafenib	SLC7A11	DUXAP8 enhances SLC7A11 activity and suppresses ferroptosis by promoting the palmitoylation of SLC7A11 and preventing its lysosomal degradation.	125
Lung cancer	KRT17P3	Non-small cell lung cancer tissue, A549, SK-MES-1, mouse models	Cisplatin	miR-497-5p, mTOR	The KRT17P3/miR-497-5p/mTOR signaling axis regulates non-small cell lung cancer chemosensitivity. Overexpression of KRT17P3 in non-small cell lung cancer cells increases cell viability and decreases apoptosis after cisplatin treatment, both *in vitro* and *in vivo*, suggesting a potential therapeutic target for patients with cisplatin-resistant non-small cell lung cancer.	128
	SFTA1P	Laryngeal squamous cell carcinoma tissues, NCl-H226, SK-MES-1, NCl-H1299, A549, A549-DDP	Cisplatin	GADD45A, hnRNP-U	SFTA1P enhances cisplatin-induced apoptosis by regulating the hnRNP-U/GADD45A pathway in laryngeal squamous cell carcinoma.	133
	MT1DP	Non-small cell lung cancer tissue, A549, H1299, mouse models	Erastin, ferroptosis	miR-365a-3p, NRF2, malondialdehyde, reactive oxygen species, glutathione	The MT1DP/miR-365a-3p/NRF2 axis contributes to the increase of erastin-induced ferroptosis in non-small cell lung cancer. Moreover, ectopic MT1DP up-regulates reactive oxygen species levels, increases intracellular ferrous iron concentration, and reduces glutathione levels in erastin-exposed cancer cells.	146
	DUXAP10	Non-small cell lung cancer tissue, PC9 cell line, mouse models	Gefitinib	EZH2, OSA2	The overexpression of DUXAP10 contributes to gefitinib resistance in non-small cell lung cancer cells and tissues by recruiting EZH2 to inhibit OAS2 expression. This forms a promising DUXAP10-EZH2-OAS2 regulatory axis to overcome acquired gefitinib resistance in non-small cell lung cancer.	142
Breast cancer	CYP4Z2P	MCF-7, mouse models	Tamoxifen	CDK3, PI3K/ERK1/2 pathways	The ceRNET between CYP4Z1 and the CYP4Z2P pseudogene functions as a sub-ceRNET that enhances CDK3 expression in estrogen receptor-positive breast cancer. This interaction represents a potential therapeutic target for treating tamoxifen-resistant breast cancer.	147
	PTENP1	Breast cancer tissues, MDA-MB-231, T-47D, MCF-7, mouse models	Adriamycin	miR-20a/PTEN/PI3K/AKT pathway	The PTENP1/miR-20a/PTEN axis is involved in the malignant behavior of breast cancer cells through the PI3K/Akt pathway.	154
	FTH1P3	Breast cancer tissue, MCF-7, MDA-MB-231, MDA-MB-468, MDA-MB-453, MCF-10A, mouse models	Paclitaxel	miR-206/ABCB1	The surveillance mechanism of FTTH1P3 in breast cancer resistance to paclitaxel through miR-2016/ABCB1 presents a new perspective for breast cancer chemotherapy.	157
Gastric cancer	SUMO1P3	Gastric cancer tissue, RGM-1, GES-1, MGC-803, BGC-7901, MKN-45, HS-746T	Cisplatin, 5-fluorouracil	CNBP, CCND1	SUMO1P3 promotes the proliferation, invasion, and drug resistance of gastric cancer cells by interacting with CNBP, highlighting a potential prognostic biomarker and a novel therapeutic target for gastric cancer.	164
	DUSP5P1	Gastric cancer tissue, AGS, SGC7901, BGC823, HGC27, MGC803, NCI-N87, MKN45, MKN74	Oxaliplatin	ARHGAP5, MAPK signaling pathways	DUSP5P1 promotes gastric cancer metastasis by directly modulating ARHGAP5 expression to activate focal adhesion pathways, with MAPK serving as a therapeutic target for platinum drug resistance.	168
Multiple myeloma	PDIA3P	U266 cells	Bortezomib	G6PD, c-Myc, pentose phosphate pathway	PDIA3P regulates multiple myeloma cell growth and bortezomib resistance through G6PD and the pentose phosphate pathway. PDIA3P acts as a novel c-Myc-interacting lncRNA, elucidating crucial roles in the metabolic regulation of multiple myeloma and offering a potential therapeutic target for patients with multiple myeloma.	171
	PTENP1	Multiple myeloma tissue, A375, A375PR1, A375VR3, A375VR4,	BRAFi, vemurafenib	CEBPβ, EZH2, PTEN, DNMT3A	PTENP1-AS is induced by the transcription factor CEBPB in BRAFi-resistant melanoma cell lines, leading to transcriptional suppression of PTEN through recruitment of EZH2 and the formation of H3K27me3 and DNA methylation of the PTEN promoter.	84
Renal cell carcinoma	PTENP1	Renal cell carcinoma tissue, 786-O, ACHN, SN12PM6	Cisplatin	miR-21, PTEN	PTENP1/miR-21/PTEN acts as a competing endogenous RNA in clear-cell renal cell carcinoma to inhibit cancer progression.	186
Ovarian cancer	SDHAP1	Ovarian cancer tissue, SKOV3, Hey-8	Paclitaxel	miR-4465, EIF4G2	The SDHAP1/miR-4465/EIF4G2 regulatory network represents a potential therapeutic target for paclitaxel-resistant ovarian cancer.	179
	CTSLP8	Ovarian cancer tissues, SKOV3, A2780, mouse models	Cisplatin	PKM2, c-Myc	CTSLP8 can directly bind to PKM2 and enhance the binding of PKM2 to the promoter region of c-Myc. Additionally, the knockdown of CTSLP8 influenced the drug resistance status of ovarian cancer cells, indicating its potential as a therapeutic target for ovarian cancer.	186
Colorectal cancer	POU5F1P4	Colorectal cancer tissue, Caco2, NCI-H508	Cetuximab	AREG, CKB, EREG, MYC, NOX1, SLC39A2, ANO1, CYP3A5, NT5E, ErbB, *etc*.	The down-regulation of POU5F1P4 reduced the sensitivity of colorectal cancer cells to cetuximab by interacting with protein-coding genes that impact various biological pathways like angiogenesis, cell surface, and epidermal growth factor receptor binding.	188
Osteosarcoma	EBLN3P	Osteosarcoma tissue, MNNG/HOS, Saos-2, U2OS, MG63	Methotrexate	miR-200a-3p, O-GlcNAc	LncEBLN3P is up-regulated in osteosarcoma and enhances methotrexate resistance in osteosarcoma cells by down-regulating miR-200a-3p. This down-regulation, in turn, promotes the epithelial-to-mesenchymal transition of osteosarcoma cells by increasing O-GlcNAc transferase.	189
Bladder cancer	PTENP1	Bladder cancer tissue, T24, 5637, mouse models	Cisplatin	miR-103a, PDCD4	Exosome-derived PTENP1 suppresses cisplatin resistance in bladder cancer by inhibiting cell proliferation and migration while promoting apoptosis through the regulation of the miR-103a/PDCD4 axis. This presents a targeted therapy option for patients with cisplatin-resistant cervical cancer.	187
Cervical cancer	ANXA2P2	Cervical cancer tissue, Caski, HeLa, mouse models	Cisplatin	miR-361-3p, SOX9	The SOX9/lncRNA ANXA2P2/miR-361-3p/SOX9 regulatory loop influences cervical cancer cell growth and resistance to cisplatin.	190
Brain cancer	PDIA3P1	Glioma cell lines U118MG, U87MG, LN229, U251MG, mouse models	Temozolomide, nefllamapimod	CEBPβ, MDM2	PDIA3P1 promotes proneural-to-mesenchymal transition by disrupting the C/EBPβ/MDM2 complex and inhibiting the ubiquitination of C/EBPβ, resulting in stronger temozolomide therapy resistance in glioma cells.	191

## Hepatocellular carcinoma

Liver cancer is the sixth most frequently diagnosed cancer globally and the fourth leading cause of deaths related to cancer.^2^ An analysis of developing countries' data indicates that over 50% of liver cancer diagnoses stem from hepatocellular carcinoma (HCC) associated with the hepatitis B virus (HBV), highlighting a pressing worldwide health concern.^107^ Despite extensive research on diagnosis and therapy, HCC has the second-lowest 5-year survival rate.^108^ HBV replication has been associated with drug resistance in HCC by activating various cancer-related signaling pathways and influencing cellular metabolism, stemness, and other mechanisms. Studies indicate that dysregulated lncRNAs linked to HBV-associated HCC play critical roles in determining disease susceptibility and clinical outcomes. These lncRNAs contribute to HCC progression through several molecular mechanisms, including transcriptional regulation, protein stabilization, epigenetic modifications, acting as molecular sponges, controlling alternative splicing, and serving as precursors for microRNA production. By engaging in these processes, dysregulated lncRNAs not only promote treatment resistance but also enhance HBV replication, further advancing HCC progression.^107^^,^^109^^,^^110^ Consistent with these findings, Xiao and colleagues demonstrated that the elevated expression of lncRNA HANR (HCC-associated long non-coding RNA) played a role in the progression of HCC. Their research highlights lncRNA HANR as a potential therapeutic target for enhancing the sensitivity of HCC cells to doxorubicin. This effect is achieved through HANR's interaction with glycogen synthase kinase 3 beta (GSK3β) interaction protein (GSKIP), which regulates the phosphorylation of GSK3β, as observed in both Hep3B cell lines and HCC patients. Furthermore, analysis of the Hep3B cell line, which is positive for HBV, suggests that viral infection may up-regulate HANR expression in HCC patients. This up-regulation could potentially drive increased cell proliferation, tumor growth in xenograft or orthotopic models, and reduced apoptosis, contributing to doxorubicin resistance. However, these findings remain speculative and require further investigation for confirmation.^109^

A recent study by Lin et al (2024) found that overexpression of the pseudogene-derived lncRNA LOC344887 enhanced cellular migration and invasion in HCC. This occurs through the phosphorylation of signal transducer and activator of transcription 3 (STAT3) at tyrosine 705, a crucial step for maintaining STAT3 activation in HCC. Additionally, it was discovered that LOC344887 regulated high-mobility group AT-hook 2 (HMGA2) expression by facilitating STAT3 binding to its promoter, which was amplified by the LOC344887/src homology region 2 domain-containing phosphatase 1 (SHP-1)/STAT3 axis. This highlights the potential of targeting this axis for therapeutic intervention in HCC.^111^ A further analysis by Lee and his colleagues has discovered that the pseudogene-derived lncRNA pleckstrin homology domain containing A8 pseudogene 1 (PLEKHA8P1) acts as a ceRNA for specific miRNAs, such as miR-516-b and miR-4766-5p. These miRNAs normally inhibit the expression of the parental gene PLEKHA8.^108^ However, the interaction between PLEKHA8P1 and these miRNAs leads to the up-regulation of PLEKHA8 expression. This up-regulation, in turn, enhances tumor progression by activating the Wnt/β-catenin signaling pathway.^108^ The research emphasizes the potential of PLEKHA8P1 as a novel biomolecular target for developing therapies to combat chemoresistance in liver cancer patients. Moreover, PLEKHA8P1 overexpression enhances proliferation, invasion, migration, and 5-fluorouracil resistance in HCC cells.^108^

Previous research has indicated that the lncRNA called protein disulfide isomerase family A member 3 pseudogene 1 (PDIA3P1) is also overexpressed in HCC. Additionally, it has been shown that this lncRNA is also up-regulated in different cancer types, and its expression is increased following exposure to DNA-damaging chemotherapy drugs, such as doxorubicin.^112^ Specifically, elevated levels of the lncRNA PDIA3P1 were linked to poorer recurrence-free survival outcomes in patients with HCC. Further experimental studies, involving both overexpression and knockdown of lncRNA PDIA3P1, revealed that this lncRNA protected cancer cells from undergoing doxorubicin-induced apoptosis, thereby enabling tumor xenografts to grow more rapidly and become more resistant to doxorubicin treatment.^112^ From a mechanistic perspective, the miR-125a/b and miR-124 were shown to inhibit the expression of the tumor necrosis factor receptor-associated factor 6 (TRAF6) protein.^112^ However, the lncRNA PDIA3P1 was shown to interact with miR-125a/b and miR-124, counteracting their inhibitory effects on TRAF6 expression. This, in turn, resulted in the activation of the nuclear factor kappa B (NF-κB) signaling pathway.^112^ Research into the mechanisms behind the up-regulation of PDIA3P1 found that the human homologue of mRNA transport mutant 4 (hMTR4), which aids in RNA degradation, could bind to PDIA3P1.^112^ Doxorubicin treatment disrupted this interaction. An increase in hMTR4 levels diminished the doxorubicin-induced rise in PDIA3P1 levels, whereas silencing hMTR4 led to an increase in PDIA3P1 levels.^112^ These results suggest that doxorubicin may boost PDIA3P1 expression by inhibiting the hMTR4-mediated degradation of PDIA3P1.^112^

A recent study has indicated a positive correlation between drug resistance, cancer stem cell-related traits, and unfavorable survival rates.^30^ In line with this finding, the pseudogene lipoprotein(A) like 2 (LPAL2) plays a significant function in reducing resistance to doxorubicin and cancer stem cell characteristics.^113^ Silencing the PDIA3P1 in liver cancer cells reduced doxorubicin's effectiveness in triggering cell death, as indicated by alterations in the expression of apoptosis-related markers. Additionally, it promoted sphere formation by overexpression of nanog homebox (Nanog, which encodes an NK2-family homeobox transcription factor), sex determining region Y-box 2 (SOX2), and lin-28 homolog A (LIN28A).^113^ Interestingly, down-regulation of the PDIA3P1 was observed in CD133-negative liver cancer cells, suggesting its potential involvement in regulating cancer stem cell functions. These connections can help explain the poorer survival outcomes observed in HCC patients with lower levels of PDIA3P1.^113^ Previous research has demonstrated that doxorubicin treatment can increase the expression of the matrix metalloproteinase-9 (MMP-9) protein in H9c2 rat heart-derived embryonic myocytes,^114^ and this effect is mediated by the p38 kinase signaling pathway. Additionally, the NF-κB transcription factor has been demonstrated to increase MMP9 expression and provide resistance to the chemotherapy drug 5-fluorouracil in colorectal cancer cell lines.^115^ The indirect connections identified imply that depleting the lncRNA PDIA3P1 triggers the expression of the MMP9 protein, contributing to doxorubicin resistance in HCC cell lines. Additional studies are necessary to clarify the direct effect of PDIA3P1 on MMP9 expression at the transcriptional level.^113^ MMP9 is a renowned driver of metastasis crucial for cancer advancement.^116^ Furthermore, the researchers have demonstrated that LPAL2 controls MMP9 at the transcriptional level. Correspondingly, increased LPAL2 levels and decreased MMP9 levels correlated with improved prognosis in HCC patients.^113^ In conclusion, these investigations indicate that LPAL2 influences the growth, invasion, migration, and stemness characteristics of HCC cell lines.^113^

A recent study unveiled a novel signaling pathway that regulates proliferation, migration, EMT, and chemoresistance in HCC by modulating the expression of forkhead box M1 (FOXM1). This pathway involves dual homeobox A pseudogene 8 (DUXAP8), miR-485-5p, and FOXM1.^117^ Notably, the study highlighted DUXAP8's role in conferring resistance to poly ADP ribose polymerase (PARP) inhibitors like olaparib in HCC cells. It also elucidated a key mechanism underlying DUXAP8 enhancing the expression of the FOXM1 transcription factor, either by sequestering the miR-485-5p or by interacting with the Fusion (FUS) protein.^117^ Functionally, the increased expression of the FOXM1 transcription factor, driven by the up-regulation of DUXAP8, significantly contributed to the oncogenic characteristics of DUXAP8. This included promoting the proliferation and migration of HCC, also conferring resistance to PARP inhibitor treatments.^117^ FOXM1, a transcription factor, exhibits diverse oncogenic functions in human cancers, encompassing proliferation, apoptosis, migration, EMT, angiogenesis, metastasis, and drug resistance.^118^ In support of these findings, a previous study by Fang et al (2015) reported that the PARP inhibitor olaparib induced the expression and nuclear localization of the FOXM1 transcription factor. This, in turn, led to the up-regulation of other homologous recombination repair genes, such as breast cancer type 1 (BRCA1) and DNA repair protein RAD51 homolog 1 (RAD51), contributing to the development of adaptive resistance to olaparib treatment.^119^ Apart from FOXM1, other factors such as EGFR, mesenchymal–epithelial transition factor (c-MET) heterodimer,^120^ p53 binding protein 1 (53BP1),^121^^,^^122^ and Schlafen family member 11 (SLFN11)^123^^,^^124^ also play a role in regulating resistance to PARP inhibitors. Therefore, future studies could explore whether DUXAP8 operates via alternative mechanisms to modulate HCC resistance to PARP inhibitors.^117^ These studies indicate the existence of mechanisms resistant to PARP inhibitors in various types of cancer. Elucidating these resistance mechanisms will not only help predict the treatment response of HCC patients to PARP inhibitors but also facilitate the development of therapies that can enhance the sensitivity of cancer cells to PARP inhibitor drugs.^117^ Moreover, recent studies have shown that increased levels of DUXAP8 in liver cancer are associated with more aggressive tumor characteristics and poorer patient outcomes. This is largely because DUXAP8 contributes to resistance against the drug sorafenib and enhances the proliferation, migration, and invasion of tumor cells.^125^ Research has indicated that DUXAP8 can diminish the sensitivity of HCC to ferroptosis induced by sorafenib, a type of programmed cell death, by influencing the expression of the solute carrier family 7 member 11 (SLC7A11) gene.^125^ On the other hand, DUXAP8 has been found to enhance the activity of SLC7A11 protein and suppress ferroptosis by promoting the palmitoylation of SLC7A11 and preventing its degradation in the lysosome.^125^ Cystine, a crucial substrate, is primarily transported into tumor cells by SLC7A11 protein within the tumor microenvironment. This cystine uptake contributes to the synthesis of glutathione, which helps tumor cells resist ferroptosis and promotes tumor growth and progression. Consequently, the down-regulation of SLC7A11 represents a promising avenue for drug discovery in diseases related to ferroptosis.^126^ This finding suggests that lncRNA DUXAP8 can improve treatment efficacy in progressive HCC patients by inhibiting the depalmitoylation of SLC7A11 and reducing its degradation to prevent ferroptosis.

## Lung cancer

Lung cancer is the leading cause of cancer-related deaths worldwide for both men and women, primarily due to its high incidence and mortality rates.^2^ NSCLC represents 85% of all lung cancer instances. Currently, the standard treatment for individuals with advanced NSCLC is platinum-based combination chemotherapy.^127^ Cisplatin, combined with a new therapeutic agent, is the most commonly used first-line chemotherapy regimen for NSCLC. However, patient responses to this treatment can differ, and the development of drug resistance greatly restricts its long-term effectiveness. Therefore, it is essential to understand the molecular mechanisms that contribute to cisplatin resistance in lung cancer.^128^

Previous studies have found that the keratin 17 pseudogene 3 (KRT17P3) is overexpressed in lung cancer tissues from patients who are resistant to cisplatin.^128^ In line with these findings, a recent study reported that plasma levels of KRT17P3 were elevated in cisplatin-resistant patients, correlating with a poor response to chemotherapy. Furthermore, functional studies have demonstrated that the overexpression of KRT17P3 in cultured NSCLC cells improves cell viability and decreases apoptosis after cisplatin treatment, both *in vitro* and *in vivo*.^128^ Conversely, silencing KRT17P3 produces the opposite effect. Mechanistically, bioinformatics analyses, RNA immunoprecipitation, and dual luciferase reporter assays have shown that KRT17P3 acts as a molecular decoy, binding to miR-497-5p and alleviating its inhibitory effect on its target gene, the mammalian target of rapamycin (mTOR).^128^ Other research has shown a decrease in miR-497-5p levels in various tumors implicated in processes related to malignancy, such as cancer initiation, progression, metastasis, and sensitivity to chemotherapy.^126^^,^^128^ Experimental studies have confirmed the functional relationship between KRT17P3, miR-497-5p, and mTOR.^128^ The overexpression of mTOR, a serine–threonine protein kinase, has been noted in multiple cancer types and is associated with tumor progression and poor patient outcomes, playing a key role in the regulation of drug resistance mechanisms.^129^^,^^130^ Collectively, the KRT17P3/miR-497-5p/mTOR regulatory axis affects the chemosensitivity of NSCLC, indicating that this axis represents a promising curative target for NSCLC patients who have developed resistance to cisplatin. Additionally, KRT17P3 could potentially serve as a peripheral blood-based biomarker for identifying NSCLC patients with cisplatin resistance.^128^

Furthermore, the lncRNA surfactant-associated 1 pseudogene (SFTA1P) has been demonstrated to enhance cisplatin-induced apoptosis by modulating the heterogeneous nuclear ribonucleoprotein U (hnRNP-U)-DNA damage-inducible protein GADD45 alpha (GADD45A) pathway in laryngeal squamous cell carcinoma. Some studies have found that SFTA1P can bind to and up-regulate hnRNP-U, also known as scaffold attachment factor (SAF)-A.^131^, ^132^, ^133^ HnRNP-U is involved in the processing and transport of mRNA molecules. Moreover, knocking down hnRNP-U contributes to cisplatin resistance by decreasing the expression of the apoptosis and DNA repair-related gene GADD45A, similar to the effects seen when SFTA1P is depleted.^131^^,^^132^ For example, hnRNP-U can increase the expression of GADD45A by stabilizing its mRNA transcript. Recent evidence indicates that hnRNP-U is involved in a range of pathological and physiological processes, including cell apoptosis,^134^ DNA damage response,^135^^,^^136^ cell viability,^137^ and RNA stability control.^138^ Furthermore, the GADD45 gene, which plays a role in DNA damage repair^139^ and the promotion of apoptosis,^140^^,^^141^ is also crucial for the survival of cancer cells.^140^ In a recent study, Ren et al (2023) have discovered that the up-regulation of the pseudooncogene-derived lncRNA double homeobox A pseudogene 10 (DUXAP10) can caused the gefitinib resistance in NSCLC cells and tissues by recruiting enhancer of zeste homolog 2 (EZH2) to the promoter region of the tumor suppressor gene 2′-5′-oligoadenylate synthetase type 2 (OAS2; a protein that binds to double-stranded RNA and plays a role in triggering the activity of RNase-L).^142^ This action results in the trimethylation of histone H3K27 (27th amino acid in histone H3) and the inhibition of OAS2 transcription. Additionally, the up-regulation of OAS2 induces apoptosis and G0/G1 phase cell cycle arrest in the gefitinib-resistant PC9/GR cell line, suggesting a promising DUXAP10-EZH2-OAS2 regulatory axis to combat gefitinib resistance in NSCLC.^142^

Ferroptosis is a unique form of cell death that is both morphologically and biochemically different from other types.^143^^,^^144^ It is marked by a reduction of glutathione and the excessive production of lipid reactive oxygen species due to the involvement of intracellular iron ions.^143^^,^^145^ Ferroptosis is recognized as a novel anti-cancer therapy. The overexpression of the nuclear factor erythroid 2-related factor 2 (NRF2), an antioxidant transcription factor, has been identified as a mechanism by which malignant cells protect themselves from ferroptosis. Previous studies have shown that the metallothionein 1D pseudogene (MT1DP), a lncRNA, exacerbates oxidative stress by inhibiting antioxidation.^146^ Consistent with this data, the overexpression of MT1DP sensitized the A549 and H1299 lung cancer cell lines to erastin-induced ferroptosis by reducing the levels of the antioxidant transcription factor NRF2.^146^ Moreover, the overexpression of MT1DP led to increased levels of malondialdehyde and reactive oxygen species, elevated intracellular ferrous iron concentrations, and reduced glutathione levels in cancer cells treated with erastin, all by sequestering miR-365a-3p.^146^ Conversely, down-regulating MT1DP produced the opposite effects. In summary, these findings highlight a new strategy to enhance erastin-induced ferroptosis in NSCLC through the MT1DP/miR-365a-3p/NRF2 regulatory axis.^146^

## Breast cancer

Patients diagnosed with estrogen receptor α (ERα)-positive breast cancer (BC) may receive endocrine therapy, such as tamoxifen. However, many of these individuals develop resistance to these treatments, which reduces their effectiveness. The exact mechanisms driving this resistance are not yet fully elucidated.^147^, ^148^, ^149^ The interaction network between cytochrome P450 family 4 subfamily Z member 1 (CYP4Z1) and the pseudogene CYP4Z2P has been identified as a promoter of breast cancer angiogenesis. Elevated levels of CYP4Z1 or CYP4Z2P-3′ untranslated region (UTR) enhance the transcriptional activity of ERα by inducing ERα phosphorylation. Moreover, the study has revealed that CYP4Z1 and CYP4Z2P-3′UTRs enhance ERα activity in a manner dependent on cyclin-dependent kinase 3 (CDK3).^147^ The findings suggest that the interaction between the CYP4Z1 gene and its pseudogene CYP4Z2P forms a sub-network that promotes the expression of CDK3 in ER-positive breast cancer. This presents a probable therapeutic target for handling tamoxifen-resistant breast cancer.^147^ Additionally, CDK3 is a target of the microRNA miR-125a-3p.^147^ Previous research has also shown that the CYP4Z2P pseudogene and the 3′UTR of the CYP4Z1 gene collectively enhance breast cancer angiogenesis by activating the PI3K and extracellular signal-regulated kinase 1/2 (ERK1/2) signaling pathways, functioning as ceRNAs for CYP4Z1.^150^ Notably, the PI3K and ERK1/2 pathways have been implicated in tamoxifen resistance.^151^ CDK3 is commonly up-regulated in cancer cell lines and tumor tissues.^152^ Specifically, CDK3 is minimally present in adjacent breast tumor tissue but shows an increase in breast cancer tissue, where it can modulate ER activity, thereby playing a role in tamoxifen resistance.^153^

Another study has found that alterations in the expression of the phosphatase and tensin homolog pseudogene 1 (PTENP1) affect breast cancer cell proliferation, invasion, tumor formation, and resistance to the chemotherapeutic drug adriamycin.^154^ Bioinformatic analysis and luciferase reporter assays indicate that PTENP1 is a direct target of miR-20a, uncovering an alternative mechanism through which PTENP1 affects the aggressive behavior of breast cancer cells. Furthermore, PTENP1 functioned as a natural inhibitor of miR-20a, regulating the expression of the PTEN tumor suppressor gene.^154^ This, in turn, controlled breast cancer cell proliferation, invasion, and drug resistance by modulating the PI3K/AKT signaling pathway. Additionally, the PI3K inhibitor LY294002 or siRNA-mediated silencing of AKT also prevented the development of breast cancer.^154^ Taken together, these findings suggest that the PTENP1/miR-20a/PTEN axis contributes to the malignant behaviors of breast cancer cells, likely through modulation of the PI3K/AKT pathway by PTEN.^154^ Consistent with the previous findings, increased expression of PTENP1 can inhibit breast cancer cell growth, metastasis, and tumor formation by suppressing miR-19b and promoting the expression of the PTEN tumor suppressor in breast cancer.^155^ Likewise, miR-20a has been shown to be abnormally expressed in breast cancer, influencing the aggressiveness of the disease through its target genes.^156^ In particular, miR-20a-5p is highly expressed in tissues and cell lines of triple-negative breast cancer, where it promotes triple-negative breast cancer cell growth by targeting the Runt-related transcription factor 3 (RUNX3). The PI3K/AKT signaling pathway, an important oncogenic pathway, is critical in the progression of various cancers, including breast cancer.^156^

Interestingly, the lncRNA ferritin heavy chain 1 pseudogene 3 (FTH1P3) was found to be elevated in breast cancer tissues and cells that are resistant to paclitaxel, in comparison to paclitaxel-sensitive tissues and parental cell lines.^157^ Experiments involving the overexpression and silencing of FTH1P3 showed that the inhibitory concentration (IC50) value of paclitaxel decreased when FTH1P3 was silenced, resulting in cell cycle arrest at the G2/M phase. In contrast, when FTH1P3 was overexpressed, the opposite effects were observed.^157^ In a xenograft mouse model, silencing FTH1P3 was found to suppress the growth of paclitaxel-resistant breast cancer cells and decrease the expression of the ABCB1 protein.^157^ Bioinformatics analyses and luciferase reporter assays confirmed that the FTH1P3 enhanced the expression of the ABCB1 protein by targeting and sequestering the miR-206, effectively acting as a miRNA “sponge”.^157^ The potential regulatory mechanism by which the FTH1P3 influences paclitaxel resistance in breast cancer, through its involvement with the miR-206/ABCB1 pathway, provides a new view that could lead to advancements in breast cancer chemotherapy.^157^ Paclitaxel is a highly effective first-line chemotherapy medication used in clinical oncology that targets and disrupts microtubules. It is derived from the Pacific yew tree (*Taxus brevifolia*) and is regarded as one of the most powerful plant-based anti-cancer agents.^158^ The molecular mechanism of paclitaxel's chemotherapeutic action is to induce cell cycle arrest at the G2/M phase and trigger cell apoptosis.^159^ ABCB1, also known as P-gp, is a well-established chemoresistance protein that contributes to multidrug resistance in cancer.^160^

## Gastric cancer

Gastric cancer is the fourth most diagnosed cancer and the third leading cause of cancer-related deaths worldwide.^161^ Patients with late-stage gastric cancer can experience substantial benefits from chemotherapy regimens that may incorporate drugs such as adriamycin, platinum-based compounds, 5-fluorouracil, vincristine, paclitaxel, and targeted therapy agents. Nevertheless, the emergence of primary or secondary drug resistance often results in treatment failure and poor prognoses for many patients with gastric cancer.^162^^,^^163^

Recent studies have revealed that an increased expression of small ubiquitin-like modifier 1 pseudogene 3 (SUMO1P3) is associated with a lower survival rate in gastric cancer.^164^ Bioinformatics analysis and RNA pull-down experiments have shown that SUMO1P3 can directly interact with cellular nucleic acid binding protein (CNBP), and that this interaction positively regulates the expression of the downstream cellular myelocytomatosis oncogene (c-myc) and cyclin D1 (CCND1).^164^ Researchers have confirmed that the lncRNA SUMO1P3 is substantially up-regulated in multiple cancer types, including colon, bladder, breast, and liver cancer. Its elevated expression of this gene is linked to tumor progression and various processes in cancer cells. The oncogenic roles of SUMO1P3 indicate that it may be a valuable prognostic biomarker and a potential therapeutic target for cancer patients.^165^ Research has indicated that c-myc is regarded as a molecular hallmark of carcinogenesis due to its frequent activation in most human tumors.^166^ In the context of c-myc's target genes, CCND1 is a notable example that acts as a crucial function in controlling the cell cycle. Aberrant levels of CCND1 can lead to malignant development by disrupting cell cycle control and promoting uncontrolled cell proliferation.^167^ The results show that SUMO1P3 promotes proliferation, invasion, and resistance to cisplatin and 5-fluorouracil in gastric cancer cells through its interaction with CNBP. This implies that SUMO1P3 has the potential to be a valuable predictive biomarker and a novel therapeutic target for gastric cancer.^164^

Furthermore, studies have shown that the up-regulation of dual specificity phosphatase 5 pseudogene 1 (DUSP5P1) is linked to reduced survival in two separate cohorts of gastric cancer patients.^168^ The study discovered that DUSP5P1 boosted the migration and invasion of gastric cancer cells during *in vitro* experiments and their metastatic potential in an animal model.^168^ Mechanistically, DUSP5P1 activates the transcription of the Rho GTPase-activating protein 5 (ARHGAP5) gene by directly binding to a TATGTG motif in the ARHGAP5 promoter region.^168^ Further analysis shows that DUSP5P1 activates the ARHGAP5 gene, which in turn stimulates focal adhesion and MAPK signaling pathways to enhance gastric cancer metastasis.^168^ Additionally, DUSP5P1 was found to obstruct the resistance pathway of platinum. Consistent with this, up-regulation of DUSP5P1 in gastric cancer cells neutralized the cytotoxic effects of oxaliplatin, while silencing DUSP5P1 combined with oxaliplatin treatment demonstrated a synergistic effect in inhibiting metastasis of gastric cancer both *in vitro* and *in vivo*.^168^ Corroborating these findings, other studies have conducted a series of functional experiments demonstrating that DUSP5P1 particularly improves the migration and invasion capabilities of gastric cancer cells *in vitro*, as well as their ability to metastasize to the lung, liver, and peritoneum *in vivo* using a nude mouse model.^168^ Taken together, these results validate the pro-metastatic role of DUSP5P1 in gastric cancer. Concurrent with its function in promoting metastasis, studies have shown that DUSP5P1 facilitates the EMT process by up-regulating key mesenchymal regulators such as β-catenin, snail, slug, and claudin-1,^168^ while down-regulating the epithelial marker E-cadherin.^169^ Evidence suggests that DUSP5P1 significantly interferes with crucial signaling pathways related to cancer progression and metastasis, particularly the focal adhesion and MAPK signaling pathways.^170^ This disruption is marked by elevated protein levels of key factors within these pathways, such as Paxillin, focal adhesion kinase (FAK), phosphorylated ERK1/2, phosphorylated p38, and MYC.^168^

## Multiple myeloma

Multiple myeloma is the second most common form of blood cancer. It is an incurable disease characterized by the buildup of malignant plasma cells in the bone marrow.^171^ Although there have been considerable advancements in understanding the mechanisms of multiple myeloma, the aspects of metabolic adaptability and drug resistance are still not well understood.^36^^,^^171^ Metabolic dysregulation stands out as a fundamental characteristic of cancer cells.^172^ A recent study investigated the lncRNA protein disulfide isomerase family A member 3 pseudogene 1 (PDIA3P), known for its high expression in multiple myeloma and association with the survival rates of patients with this disease. PDIA3P has been found to regulate multiple myeloma cell growth and resistance to the drug bortezomib through its influence on the enzyme glucose 6-phosphate dehydrogenase (G6PD) and the pentose phosphate pathway.^171^ Mechanistically, PDIA3P was shown to interact with the c-Myc transcription factor, enhancing its ability to activate the G6PD gene promoter and thereby stimulating G6PD expression and flux through the pentose phosphate pathway.^171^ Increasing evidence suggests that targeting metabolism, particularly the pentose phosphate pathway, fatty acids, and amino acids metabolism, holds promise for enhancing multiple myeloma therapy.^173^, ^174^, ^175^ While significant strides have been made in understanding how metabolic changes contribute to cancer progression, there is still a lack of knowledge regarding the basic mechanisms and regulatory processes that control metabolic adaptability in multiple myeloma.^171^ The study has uncovered a novel regulatory mechanism in multiple myeloma, where the lncRNA PDIA3P interacts with the c-Myc oncogene to modulate its transcriptional activity, thereby regulating the expression of the downstream gene G6PD and influencing flux through the pentose phosphate pathway.^171^ The c-Myc oncogene is regulated through various mechanisms, including transcriptional control, post-transcriptional modifications, and interactions with other proteins or ncRNAs.^171^^,^^176^

In a study published in 2021, researchers show that the level of the lncRNA PTENP1-AS is promoted by the transcription factor CCAAT/enhancer binding protein beta (CEBPB) transcription factor in melanoma cell lines that are resistant to B-Raf proto-oncogene, serine/threonine kinase (BRAF) inhibitors.^84^ This induction leads to the transcriptional repression of the PTEN tumor suppressor gene by recruiting the EZH2 methyltransferase, which catalyzes the formation of repressive H3K27me3 histone marks and DNA methylation at the PTEN promoter.^84^ Furthermore, the level of PTENP1-AS has been found to predict clinical outcomes in stage III melanoma patients, with high expression of this lncRNA in initial regional lymph node metastases being associated with poorer overall survival rates.^84^ In addition, PTENP1-AS plays a significant function in both the progression of melanoma tumors and the development of resistance to the BRAF inhibitor drug vemurafenib.^84^ In recent years, researchers have gained a better comprehension of the molecular mechanisms underlying acquired resistance to BRAF inhibitors in melanoma.^177^ Also, the loss of the PTEN tumor suppressor and subsequent activation of the PI3K/AKT signaling pathway have been identified as contributing factors to this process.^178^ The overall findings of the study indicate that PTENP1-AS could be an effective target for re-sensitizing melanoma cells to BRAF inhibitor therapy and may also serve as a potential prognostic marker for clinical outcomes in patients with stage III melanoma.^84^

## Ovarian cancer

Ovarian cancer is regarded as one of the top causes of illness and mortality among women globally.^161^ The chemotherapeutic resistance observed in ovarian cancer remains a significant challenge in clinical practice. Recently, there have been reports of the abnormal increased expression of the lncRNA succinate dehydrogenase complex flavoprotein subunit A pseudogene 1 (SDHAP1) in ovarian cancer cell lines that are resistant to the chemotherapy drug paclitaxel.^179^ Nevertheless, research on the regulatory role of SDHAP1 in chemotherapeutic resistance in ovarian cancer is scarce, and the precise mechanisms are still unknown. Investigations have shown that SDHAP1 is overexpressed in ovarian cancer cell lines that are resistant to paclitaxel, such as Hey-8 and SKOV3, and this increased SDHAP1 expression is associated with decreased levels of miR-4465.^179^ Moreover, inhibiting the expression of SDHAP1 was found to restore sensitivity to paclitaxel chemotherapy in ovarian cancer cells in laboratory studies.^179^ Mechanistically, SDHAP1 was shown to increase the expression of the protein eukaryotic translation initiation factor 4 gamma 2 (EIF4G2) by acting as a decoy for miR-4465, which in turn promoted paclitaxel-induced cell death in ovarian cancer cells.^179^ The regulatory network involving the interactions between SDHAP1, miR-4465, and EIF4G2 could represent a promising therapeutic target for treating ovarian cancer that has developed resistance to paclitaxel chemotherapy.^179^ Prior research has indicated a connection between the lncRNA SDHAP1 and resistance to chemotherapy drugs in ovarian cancer, even though research specifically examining this relationship remains scarce.^180^ Additionally, research has shown that miR-4465 can bind to the 3′UTR of the PTEN gene, which suppresses PTEN expression and subsequently inhibits autophagy through the AKT/mTOR signaling pathway in HeLa cervical cancer cells.^181^ Additional studies have shown that miR-4465 might inhibit tumor growth and metastasis by directly binding to the mRNA of the oncogene EZH2 in NSCLC.^182^ Additionally, eukaryotic translation initiation factor 4F (EIF4F) is a protein complex that initiates translation, comprising ATP-dependent RNA helicase EIF4A, cap-binding protein EIF4E, and scaffolding protein EIF4G.^183^ EIF4G exists as three homologous proteins (EIF4G1, EIF4G2, and EIF4G3) which have a similar structural organization but perform different biological roles.^184^ Subsequently, these findings showed that EIF4G2, a direct target of miR-4465, was increased when SDHAP1 was overexpressed as it functioned as a sponge for miR-4465. The functional analysis indicated that the SDHAP1/miR-4465/EIF4G2 regulatory axis was linked to the apoptosis induced by paclitaxel in ovarian cancer. Furthermore, it has been previously reported that the up-regulation of EIF4G2 leads to a reduction in cisplatin sensitivity in NSCLC.^185^

Another study demonstrated that the cathepsin L1 pseudogene 8 (CTSLP8) was expressed at higher levels in ovarian cancer tumor tissues that were resistant to chemotherapy. However, research on the relationship between CTSLP8 expression and resistance to the chemotherapy drug cisplatin in ovarian cancer remains limited.^186^ The study discovered that CTSLP8 promoted the growth of ovarian cancer cells and heightened their resistance to cisplatin.^186^ Furthermore, CTSLP8 was found to enhance the expression of the c-Myc oncogene by aiding the binding of the pyruvate kinase M2 (PKM2) protein to the promoter region of c-Myc, which subsequently increased glycolysis. Analysis of tissue samples showed that increased expression of CTSLP8 was linked to the development of cisplatin resistance and poor clinical outcomes in ovarian cancer patients.^186^ These results suggest that CTSLP8 interacts with PKM2 to regulate the c-Myc oncogene, resulting in increased cellular glycolysis and contributing to the progression of ovarian cancer and the development of chemotherapy resistance. Consequently, CTSLP8 could represent a potential therapeutic target for ovarian cancer.^186^

## Other cancer

Pseudogene-derived lncRNA is important for controlling how cancer cells respond to drugs. When the regulation of this lncRNA is disturbed by harmful signals, it can lead to cellular abnormalities and even the formation of tumors. Understanding these mechanisms can provide insights into potential targets for improving cancer therapy strategies. In clear-cell renal cell carcinoma (ccRCC), research has revealed that PTENP1 is expressed at lower levels in both ccRCC tissue samples and cell lines.^187^ This reduced expression of PTENP1 is due to a process called methylation. Additionally, PTENP1 and PTEN are directly targeted and suppressed by miR-21 in ccRCC cell lines. The expression of miR-21 enhances cell proliferation, migration, and invasiveness in ccRCC cell cultures.^187^ Additionally, elevated miR-21 levels promote tumor growth and metastasis in animal models of ccRCC. Conversely, when the PTENP1 is overexpressed in ccRCC cells, it makes these cells more sensitive to the chemotherapeutic drugs cisplatin and gemcitabine, both in cell culture experiments and in animal studies.^187^ Moreover, patients with high levels of PTENP1 or PTEN show notably better overall survival rates than those with lower expression of these lncRNAs. This indicates that PTENP1 and PTEN might serve as valuable biomarkers for forecasting the prognosis and clinical outcomes in patients with ccRCC.^187^ Furthermore, lncRNA POU class 5 homeobox 1 pseudogene 4 (POU5F1P4) was found to be down-regulated in cells that developed resistance to cetuximab, as well as in metastatic colorectal cancer patients who were resistant to cetuximab treatment. Down-regulation of lncRNA POU5F1P4 led to a decrease in cetuximab sensitivity of colorectal cancer cells.^188^ The results suggest that lncRNA POU5F1P4 may play a role in conferring resistance to cetuximab by interacting with protein-coding genes involved in different cellular functions. These processes include the formation of new blood vessels (angiogenesis), cell surface functions, and the epidermal growth factor receptor binding.^188^ A study by Sun et al has discovered that osteosarcoma cells with higher lncRNA endogenous bornavirus-like nucleoprotein 3 (EBLN3P) expression exhibit resistance to methotrexate treatment.^189^ The researchers found that down-regulating LncEBLN3P reduced the resistance of osteosarcoma cells to methotrexate. This effect was mediated by the lncRNA sponging (sequestering) miR-200a-3p, a microRNA that influences the EMT process.^189^ The reduced expression of miR-200a-3p led to elevated levels of its target gene, O-GlcNAc transferase (OGT). This increase in OGT subsequently enhanced the EMT process in osteosarcoma cells.^189^ Analysis of serum exosomes from bladder cancer patients who did not respond to cisplatin treatment revealed that the expression of the PTENP1 gene was lower compared with patients who responded to the therapy. Introducing PTENP1 into cisplatin-resistant bladder cancer cells through transfection inhibited their proliferation and migration, enhanced apoptosis, and decreased their resistance to cisplatin. The study demonstrates that PTENP1 regulates various cellular processes, including cell viability, migration, apoptosis, and cisplatin resistance, in cisplatin-resistant bladder cancer cells.^187^ This is achieved through the interaction of PTENP1 by the miR-103a/programmed cell death protein 4 (PDCD4) axis. The relationship between PTENP1 and the miR-103a/PDCD4 pathway offers a promising targeted therapy option for patients with bladder cancer that is resistant to cisplatin.^187^ Research has revealed a regulatory loop involving the SRY-box transcription factor 9 (SOX9) gene/lncRNA annexin A2 pseudogene 2 (ANXA2P2)/miR-361-3p/SOX9 regulatory axis, which impacts cell growth and resistance to the chemotherapeutic drug cisplatin in cervical cancer.^190^ The levels of miR-361-3p were found to be lower in cervical cancer cells and tissue samples that were resistant to cisplatin. This decrease in miR-361-3p expression facilitated the growth of cisplatin-resistant cervical cancer cells and increased their resistance to cisplatin.^190^ On the other hand, the expression of the lncRNA ANXA2P2 was increased in cisplatin-resistant cervical cancer cells and tissue samples. This lncRNA inhibited the expression of miR-361-3p by directly interacting with it, which in turn led to the up-regulation of the transcription factor SOX9.^190^ Furthermore, SOX9 levels were found to be higher in cervical cancer cells and tissues that were resistant to cisplatin, and SOX9 promoted the transcription of lncRNA ANXA2P2 by binding to it.^190^ New research has uncovered that the overexpression of lncRNA PDIA3P1 is linked to the glioblastoma temozolomide resistance in cancer cell lines. This lncRNA has been found to be highly expressed in the mesenchymal subtype of glioblastoma.^190^ Furthermore, PDIA3P1 promotes the proneural-to-mesenchymal transition in glioma stem cells, thereby increasing the resistance of glioblastoma to temozolomide treatment. This resistance is due to the disruption of the CCAAT enhancer binding protein beta (C/EBPβ)-MDM2 complex caused by PDIA3P1.^190^ Moreover, neflamapimod is a small-molecule drug that specifically targets the p38α protein and can effectively cross the blood–brain barrier. Neflamapimod has been shown to block the temozolomide-induced increase of PDIA3P1 and exhibits synergistic effects when used alongside temozolomide at certain concentrations.^190^ The combination of temozolomide and neflamapimod has demonstrated excellent synergistic anti-tumor effects both in cell culture experiments and animal studies. This discovery may pave the way for new treatment approaches for glioblastoma patients who have developed resistance to temozolomide.^191^

## Concluding remarks and future directions

Cancer continues to be a major cause of mortality worldwide, with chemotherapy being a primary treatment option for many patients. The development of innovative molecularly targeted drugs has substantially enhanced the effectiveness of cancer therapy and prolonged patient survival. However, drug resistance remains a significant obstacle in the management of cancer.^18^ Recently, significant research has focused on understanding the mechanisms of drug resistance in cancer cells and creating strategies to overcome this resistance. Researchers have identified several protein-coding genes, including multidrug resistance mutation (MDR1), ABCG2, and MRP, that play essential roles in drug resistance. Some of these genes have been utilized in developing treatment approaches for cancer patients.^192^ Moreover, ncRNAs, including miRNAs and lncRNAs, have been linked to the development of drug resistance in cancer cells. These lncRNAs play a critical role in regulating gene expression, and their dysregulation has been linked to various diseases, such as cancer. Understanding how these lncRNAs contribute to drug resistance may lead to the creation of new treatment strategies for cancer patients.^193^^,^^194^

Initially, pseudogenes were thought to be genomic elements without protein-coding capabilities, deemed “nonfunctional” due to inactivating mutations. Although pseudogenes are evolutionarily conserved and found in both prokaryotes and eukaryotes, they have frequently been regarded as genetic “junk” or “relics”.^94^ The development of high-throughput sequencing techniques, especially omics-based technologies, has significantly enhanced our understanding of pseudogenes.^94^ Contrary to previous beliefs, pseudogenes have been found to possess diverse functions at the DNA, RNA, and protein levels. They actively participate in gene regulation, affecting the development and progression of various diseases, particularly cancer.^94^ Surprisingly, some pseudogenes have been shown to generate proteins, challenging their previous classification as non-functional “junk DNA” or “trash”. These pseudogenes display expression patterns that are specific to certain tissues and disease subtypes, highlighting their potential importance in disease diagnosis.^94^ Moreover, pseudogenes have been linked to patient survival rates and show significant promise for future applications in disease therapy, suggesting their value as biomarkers and targets for treatment in clinical settings.^94^ lncRNAs transcribed from pseudogene locations can regulate gene transcription and epigenetic mechanisms. These lncRNAs can act as guides, binding partners, or ceRNAs to sequester miRNAs.^195^

According to reports, lncRNAs derived from pseudogenes are frequently disrupted in different diseases. Yet, the roles of most lncRNAs derived from pseudogenes remain ambiguous. Understanding their functions is complicated by their limited expression levels and specific tissue distribution.^88^ Typically, research on lncRNAs derived from pseudogenes begins with the genes corresponding to the pseudogenes. The substantial similarity between lncRNAs derived from pseudogenes and transcripts from their corresponding genes can complicate efforts to explore the functions of these lncRNAs.^88^

As per the discussed studies, pseudogene-derived lncRNAs significantly impact gene expression and signaling pathways associated with drug resistance. This involvement leads to various functions such as cell cycle regulation, DNA repair, cell proliferation, EMT, metastasis, apoptosis, autophagy, drug efflux transporters, epigenetic modifications (*e.g.*, miRNA sponging), and the generation of cancer stem cells, playing a figure in the emergence of drug resistance in cancer therapy.^101^^,^^196^ Most research on how pseudogene-derived lncRNAs function at the molecular level has focused on the ceRNA hypothesis. This proposes that these lncRNAs compete for shared miRNAs with either their parent genes or other unrelated genes.^196^ Pseudogene-derived lncRNAs, a specific type of lncRNA, can influence cancer regulation through other means as well, such as binding to transcription factors, producing miRNAs or PIWI-interacting RNAs (piRNAs), and possibly encoding proteins.^88^

In summary, dysregulation in the expression and/or role of lncRNA transcripts originating from pseudogenes has been linked to several diseases, including cancer and drug resistance. Therefore, an in-depth analysis of the expression patterns, functions, and molecular mechanisms of pseudogene-derived lncRNAs in human cancers could be essential for developing effective strategies to combat drug resistance in cancer treatment. Moving forward, further research is necessary to confirm the findings on the functions and mechanisms of known pseudogene-derived lncRNAs. This will involve conducting both basic laboratory experiments and large-scale clinical trials. Additionally, discovering new pseudogene-derived lncRNAs, understanding their functions in different cancer types and subtypes, and creating strategies based on these transcripts for diagnosis, treatment, and prognosis present promising opportunities for advancing cancer care. By targeting these lncRNAs, it may be possible to inhibit the onset and progression of cancer, improve patient outcomes, and increase the effectiveness of cancer treatments. Consequently, ongoing research in this field is essential for expanding our knowledge of cancer biology and creating new personalized treatments for this devastating disease.

## CRediT authorship contribution statement

**Mahsa Aghajani Mir:** Writing – review & editing, Writing – original draft, Visualization, Validation, Supervision, Resources, Project administration, Investigation, Data curation, Conceptualization. **Abdolreza Daraei:** Writing – review & editing, Validation, Conceptualization.

## Conflict of interests

The authors confirm that there is no conflict of interests to report.
